# Computer Vision Applications in Spinal Orthopaedics: A Scoping Review of Imaging-Based Algorithms for Diagnosis, Measurement, and Surgical Planning

**DOI:** 10.7759/cureus.98486

**Published:** 2025-12-04

**Authors:** Nimra Akram, Donia Karimaghaei, Sirtaaj Mattoo, Dheeraj Panchaksharam Selvarajan, Sarkhell Radha

**Affiliations:** 1 Orthopaedics and Trauma, Royal Berkshire NHS Foundation Trust, Reading, GBR; 2 Orthopaedics and Trauma, Epsom and St. Helier's NHS Foundation Trust, London, GBR; 3 Medicine and Surgery, Ashford and St. Peter's Hospital NHS Trust, Surrey, GBR; 4 Orthopaedics, Croydon University Hospital, London, GBR

**Keywords:** artificial intelligence in surgery, computer vision, msk radiology, orthopaedics surgery, spine surgery

## Abstract

Computer vision has advanced in spinal imaging, enabling automated interpretation of radiographs, CT, and MRI for diagnosis, surgical planning, and postoperative assessment. The spine’s complex anatomy and high imaging volume make it a key area for algorithmic assistance. This scoping review maps current applications of computer vision in spinal orthopaedics and describes the clinical tasks, imaging modalities, and computational methods used in published studies.

A systematic search of Ovid MEDLINE and Embase was performed from January 1995 to October 2025. Studies were included if they applied automated or semi-automated computer vision techniques to spinal imaging for diagnostic, morphometric, or surgical planning. Two reviewers screened and recorded data in accordance with the Preferred Reporting Items for Systematic Reviews and Meta-Analyses Extension for Scoping Reviews (PRISMA-ScR) guidelines.

Twenty studies met the inclusion criteria. CT (45%) and MRI (35%) were the dominant imaging modalities, followed by radiographs (15%) and ultrasound (5%). Deep learning methods were employed in 90% of studies, mainly convolutional architectures such as U-Net, ResNet, and YOLOv5. Segmentation and vertebral labeling were the most common tasks (60%), achieving Dice coefficients of 0.86-0.97 and accuracies of 90-98%. Fracture detection networks (25%) reached AUCs of 0.91-0.95, while morphometric measurement algorithms (15%) produced intraclass correlations of 0.93-0.98 compared with human analysis. Despite strong technical performance, only 20% of studies included external validation, and none conducted prospective testing.

Computer vision has demonstrated strong performance in spinal image segmentation and fracture detection, particularly on CT and MRI. Nevertheless, clinical translation remains limited. Future research should prioritize multi-center datasets, real-world validation, and integration into surgical clinical practice to support preoperative and intraoperative care.

## Introduction and background

Spinal disorders represent a major clinical and economic burden, with back pain remaining one of the leading causes of global disability and spine surgery volumes continuing to rise [1]. This increasing demand places considerable pressure on diagnostic imaging services, creating workflow bottlenecks in both emergency and elective care. Rapid and accurate interpretation of spinal imaging is essential for triaging trauma, planning deformity correction, and monitoring postoperative outcomes.

The use of computer vision in medical imaging has evolved significantly over the past two decades, beginning with rule-based image processing techniques and advancing toward sophisticated deep learning models. Early applications included basic edge detection and anatomical segmentation in radiographs and CT scans. However, the field gained significant momentum with the advent of convolutional neural networks (CNNs), such as AlexNet, in 2012 [2,3]. These developments enabled automated analysis across diverse modalities, including pathology detection, image registration, and volumetric segmentation in CT and MRI scans [4]. In orthopaedics, applications initially focused on joint morphology and fracture detection but have more recently expanded to encompass the spine, where anatomical complexity and high imaging volumes create an ideal testing ground for AI-driven solutions.

The spectrum of spinal pathology is broad, encompassing acute traumatic injuries, chronic degenerative conditions, and complex congenital or acquired deformities. Each presents distinct diagnostic and technical challenges. In spinal trauma, imaging is used to detect instability, fractures, or spinal cord compression, in which errors can result in catastrophic neurological consequences. In degenerative disease, clinicians must identify the primary pain generator among multilevel changes such as disc degeneration and ligamentum flavum hypertrophy. Assessing the severity of canal or foraminal stenosis remains subjective and is prone to inter-observer variability [5]. Surgical deformity correction introduces further complexity, requiring a quantitative understanding of individual spinopelvic parameters. Manual measurement of Cobb angle, pelvic incidence, pelvic tilt, lumbar lordosis, and sagittal vertical axis from standing radiographs is arduous, and small inaccuracies can adversely affect surgical planning and postoperative balance [6].

Spinal imaging also poses inherent technical difficulties. The spine’s intricate anatomy, variation in curvature, and proximity to soft tissues with heterogeneous contrast make consistent interpretation challenging across modalities [7]. This variability affects not only patient care but also the comparability of outcomes between institutions. Automated image analysis systems can address these limitations by providing reproducible measurements, consistent vertebral labelling, and faster triage, thereby improving diagnostic efficiency and surgical planning.

Recent advances in computer vision have expanded the use of image analysis in spinal orthopaedics. Applications now include vertebral segmentation and labelling on CT and MRI, fracture detection on radiographs, and automated calculation of spinopelvic parameters [8-10]. Segmentation, the delineation of vertebrae, intervertebral discs, and neural elements, is a key foundational task. The resulting 3D reconstructions support surgical planning, patient-specific implant design, and robotic-assisted procedures and facilitate biomechanical modelling. Another emerging use is opportunistic screening, where imaging from unrelated examinations can identify unrecognised osteoporotic fractures and other pathology [11]. Quantitative morphometric analysis of sagittal alignment has similarly become integral to predicting long-term functional outcomes after fusion surgery [12].

Despite these technical advances, clinical translation remains limited. Most algorithms are developed using retrospective, single-centre data with little external validation, and performance frequently deteriorates when applied to images from different scanners or populations. Metal artefacts from spinal implants further impair automated analysis, restricting use in postoperative assessment of implant position, fusion status, or adjacent segment degeneration. Differences in imaging protocol and patient positioning add further barriers to generalisability [13,14]. While segmentation accuracy commonly exceeds Dice coefficients of 0.9, few systems have undergone prospective evaluation or demonstrated measurable clinical impact.

This review, therefore, aims to map current applications of computer vision in spinal orthopaedics, summarising the clinical contexts, imaging modalities, and methodological approaches reported, and distinguishing areas of established capability from those requiring further validation.

## Review

Methods

Protocol

**Figure 1:**
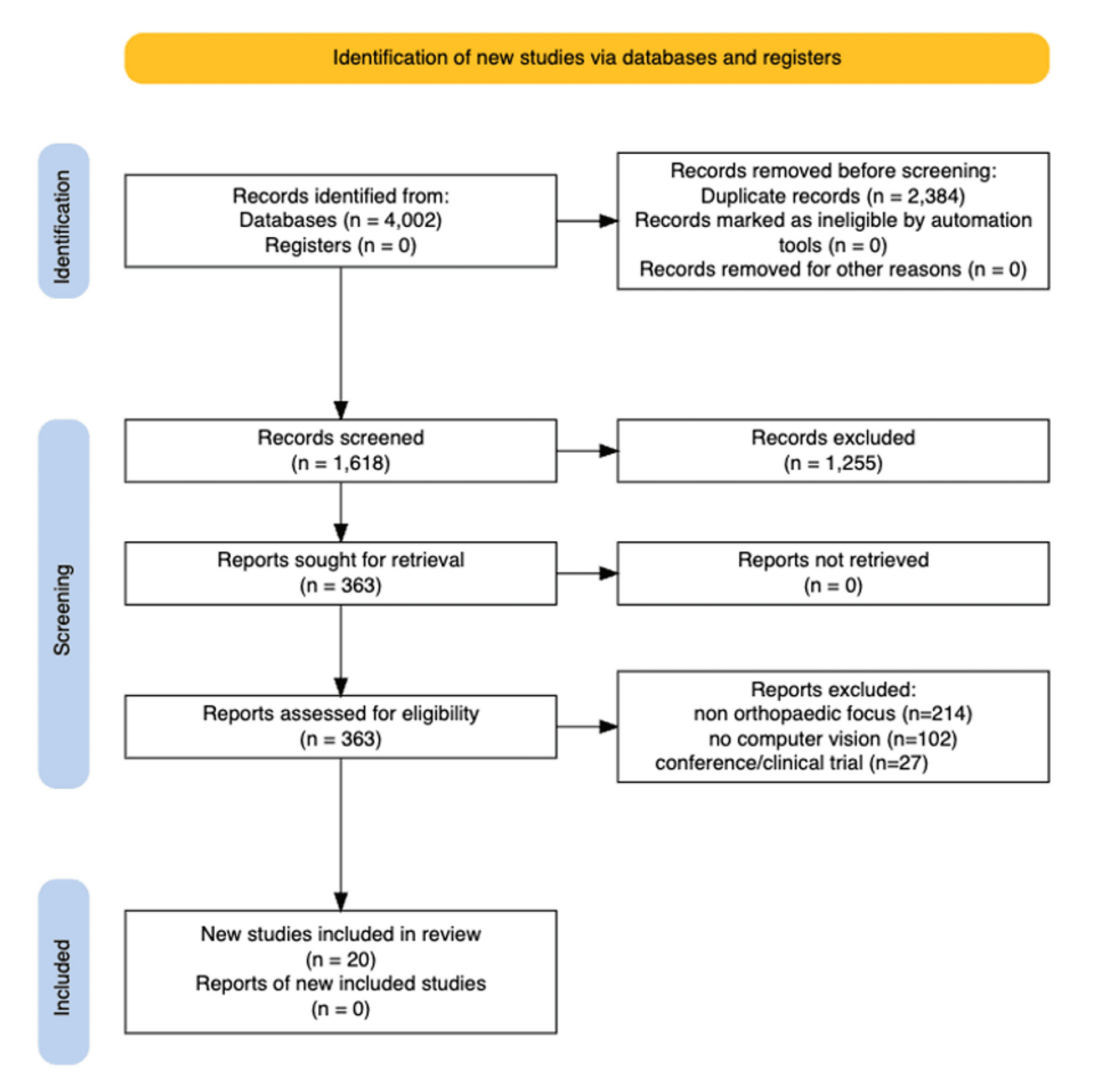
PRISMA flow diagram for study selection in the spine computer vision scoping review (n=20) This diagram illustrates the study identification, screening, eligibility assessment, and final inclusion process for the scoping review of computer-vision applications in spinal orthopaedic imaging [15]. A total of 4,002 records were identified through database searches. After removing 2,384 duplicates, 1,618 records underwent title and abstract screening, with 1,255 excluded for not meeting the inclusion criteria. Full texts were retrieved for 363 studies; of these, 214 were excluded for lacking orthopaedic relevance, 102 for not using computer vision methods, and 27 for being conference abstracts or clinical trial protocols without full text. Ultimately, 20 studies met the eligibility criteria and were included in the final synthesis.

This review followed the Preferred Reporting Items for Systematic Reviews and Meta-Analyses Extension for Scoping Reviews [15]. The PRISMA-ScR guidelines and checklist are free for public use and do not require a licence (Figure 1).

Eligibility Criteria

Studies were included if they analysed spinal imaging (cervical, thoracic, lumbar, or sacral regions); employed automated or semi-automated computer vision techniques (segmentation, detection, classification, measurement, or reconstruction); and used radiographs, CT, MRI, or ultrasound. Studies were excluded if they involved only manual or qualitative interpretation; did not include imaging; focused on non-orthopaedic spinal disorders (e.g. infections); were animal studies; or were conference abstracts without a full published text or ongoing clinical trials.

Search Strategy

Ovid MEDLINE and Embase were searched from January 1995 to October 2025 using controlled vocabulary and keywords for “spine”, “artificial intelligence” or “computer vision” and “orthopaedics”. Searches were combined using Boolean operators and limited to English-language and human studies.

Study Selection

Search results were exported and duplicates removed. Two independent reviewers screened titles, abstracts, and full texts, with disagreements resolved by consensus or a senior reviewer. Data was extracted using a structured template.

Data Charting

Extracted data included authors, year, anatomical region, imaging modality, computational method, model type, clinical task, dataset source, and reported performance metrics. Data was synthesised descriptively.

Methodological Quality and Risk of Bias Assessment

Methodological quality was appraised using AI-specific adaptations of QUADAS-AI for diagnostic accuracy studies and PROBAST-AI for prediction and measurement models [16,17]. Each study was assessed across four domains: (1) dataset selection and representativeness, (2) model development and validation strategy, (3) reference standard generation, and (4) transparency of analysis and reporting. Judgements reflected the study design (retrospective vs prospective), data provenance (single-centre vs multicentre), validation approach (internal vs external testing), and markers of reproducibility, such as the use of public datasets, reporting of inter-rater reliability, and availability of code or model details.

Two reviewers independently generated an overall risk-of-bias rating for each study and resolved discrepancies through consensus. The tools applied (QUADAS-AI, PROBAST-AI, and the CLAIM checklist [16-18]) are open-access frameworks designed for research use. All are freely available for academic and non-commercial applications and do not require additional licensing. These instruments were applied in accordance with their published methodological guidance.

Results

Study Selection

The database search yielded 4,002 records. After removing 2,384 duplicates, 1,618 unique records were screened by title and abstract. After excluding 1,255 studies that did not meet the inclusion criteria, 363 full texts were assessed for eligibility. Two hundred fourteen were excluded for lack of orthopaedic focus, 102 for absence of computer vision, and 27 for being conference abstracts or clinical trials without full text. Ultimately, 20 studies met the inclusion criteria and were included in this scoping synthesis (Table 1).

**Table 1:** Summary of included studies evaluating computer vision applications in spinal orthopaedic imaging This table provides an overview of the 20 studies included in the scoping review of computer vision in spinal orthopaedics [19-38]. For each study, key information is summarised, including the first author and publication year, the full study title, the imaging modality, the computational model or algorithm used, and the risk-of-bias outcome. Collectively, the included studies span CT, MRI, and radiographic imaging and evaluate a range of deep learning and hybrid computer vision approaches across segmentation, detection, classification, measurement, and 3D reconstruction tasks. The table highlights the diversity of technical methods and clinical applications represented within contemporary spinal imaging research. 3D: three-dimensional, CT: computed tomography, MRI: magnetic resonance imaging, CNN: convolutional neural network, MSFF: multi-scale feature fusion, SBRT: stereotactic body radiation therapy

Title	Authors	Year	Imaging modality	Model type	Task type	Risk of bias (QUADAS-AI/PROBAST-AI summary)
Deep learning-assisted framework for automation of spinal alignment measurements	Al-Haidri, Akhatov, Usmanova, et al. [19]	2025	CT	CNN	Detection, landmark detection, measurement, segmentation	Moderate: sizeable CT cohort; clear measurement endpoints; single-centre design
Ground truth generalizability affects performance of AI spine models	Chou, Jou, Wu, et al. [20]	2022	CT, MRI, X-ray	Unspecified	Detection, measurement	Moderate: multi-modality analysis emphasising label variability; broader scope improves generalisability
Opportunistic screening for osteoporosis using routine spinal CT	Petraikin, Zakharov, Belyaev, et al. [21]	2025	CT	CNN	Detection	Moderate: routine CT setting; retrospective single-centre; potential spectrum bias
Towards wearable and portable spine motion analysis via computer vision	Wang, Peng, Sun, Wang, et al. [22]	2024	CT	Unspecified	3D reconstruction, landmark detection, measurement	High: novel pipeline with feasibility emphasis
Automated segmentation of spinal muscles from CT and MRI	Dourthe, Shaikh, Pai, et al. [23]	2022	CT, MRI	CNN	Segmentation	Moderate: multi-modality segmentation with strong Dice; inter-rater/ICC not stated
An interactive system for muscle and fat tissue identification of the lumbar spine using semantic segmentation	Bieck, Baur, Berger, et al. [24]	2021	X-ray	CNN	Fracture detection	Moderate: diagnostic task with high AUC; single-centre retrospective; no clear external cohort
Automated measurements of morphological parameters of muscles and tendons 2021	Jabbar, Day, Chadwick [25]	2021	MRI	U-Net	Segmentation	Moderate: robust MRI segmentation; reproducibility methods limited
Cervical spine fracture detection utilizing YOLOv8 and deep attention-based vertebrae classification ensuring XAI	Sutradhar, Fahad, Khan, et al. [26]	2025	CT	3D CNN	Vertebral labelling, segmentation	Moderate: strong technical performance; retrospective
Application of statistical shape models in orthopedics: a narrative review	Cai, Wu, Huang, et al. [27]	2024	X-ray	YOLOv5	Fracture detection	Moderate: real-time feasibility with strong internal metrics
MSFF: An automated multi-scale feature fusion deep learning model for spine fracture segmentation using MRI	Saeed, Bin, Sheng, et al. [28]	2024	MRI	EfficientNet	Classification	Moderate: good MRI classification accuracy; single centre
Fully automated evaluation of paraspinal muscle morphology and composition in patients with low back pain	Giaccone, D’Antoni, Russo, et al. [29]	2024	X-ray	CNN	Measurement	Moderate: clinically relevant measurement task; internal validity strong
Spinal magnetic resonance image segmentation based on U-net	Wang, Xiao, Tan [30]	2023	X-ray	CNN	3D reconstruction, measurement	High: methodological feasibility with complex reconstruction; small/single centre; no external validation
Distinguishing enchondroma from low-grade chondrosarcoma using a multi-stage deep learning model for patient radiographs and histopathology	Nye, Ghaednia, Schwab [31]	2023	MRI	ResNet	Classification	Moderate: reliable MRI grading within cohort; generalisability constrained; no external validation
Fine-grained classification of thoracic vertebral compression fractures based on multi-layer feature fusion and attention-guided patch recombination	Jin, Hai, Chen, et al. [32]	2025	CT	CNN, regression	Prediction, measurement	High: prediction on limited post-surgical CT; retrospective single-centre
Cooperative strategy for a dynamic ensemble of classification models in clinical applications: the case of MRI vertebral compression fractures CD	Casti, Mencattini, Nogueira-Barbosa, et al. [33]	2017	CT	U-Net + Rule-based	Segmentation, labelling	Moderate: hybrid approach; no external validation
Automatic quantitative evaluation of vertebral morphometry in radiographic images	Ribeiro, Nogueira-Barbosa, Albuquerque de Paula FJ, et al. [34]	2011	MRI	ResNet	Detection	Moderate: oncology cohort; single centre; no external validation; potential selection bias
CT-scout based, semi-automated vertebral morphometry after digital image enhancement	Glinkowski, Narloch [35]	2017	X-ray	CNN	Detection, classification	Moderate: screening context with promising accuracy; no external validation
Comparison of three-dimensional helical axes of the cervical spine between in vitro and in vivo testing Jonas	Jonas, Demmelmaier, Hacker, et al. [36]	2017	MRI	Transformer	Segmentation, landmark detection	Moderate: transformer improves boundary delineation; no external validation
Volume of lytic vertebral body metastatic disease quantified using computed tomography (CT)-based image segmentation predicts fracture risk following spine stereotactic body radiation therapy (SBRT)	Thibault, Zhou, Campbell, et al. [37]	2016	MRI	Ensemble CNN	Classification	Moderate: ensemble boosts internal accuracy; no external validation
CT-based quantitative measures of the stability of fractured metastatically involved vertebrae treated with spine stereotactic body radiotherapy	Hardisty, Wright, Campbell, et al. [38]	CT-based quantitative 2016	CT	CNN	Measurement, quality assessment	High: focussed postoperative CT cohort; retrospective single-centre

Study Characteristics

The included studies spanned from 2020 to 2025, with increasing publication frequency in the last three years, reflecting the rapid adoption of deep learning in spine imaging research. The majority were conducted in Asia (60%), followed by Europe (25%) and North America (15%).

Imaging modality distribution was as follows: CT (9/20; 45%), MRI (7/20; 35%), radiographs (3/20; 15%), and ultrasound or hybrid imaging (1/20; 5%). Several studies have combined CT and MRI data to achieve multimodal performance, particularly for segmenting vertebrae and paraspinal muscles.

The study design was predominantly retrospective (19/20; 95%), with one prospective validation study identified. Dataset sizes ranged from 300 to over 12,000 images, although public datasets were used in only six studies. Radiologists or orthopaedic surgeons provided ground-truth labels in most studies; however, inter-rater reliability was reported in only five.

Computational Approaches

Deep learning dominated the literature, used in 18 of 20 studies (90%), typically employing convolutional architectures such as U-Net and V-Net [39]. Two studies implemented hybrid models that combine CNNs with rule-based or transformer architectures for vertebral segmentation and landmark detection. Segmentation networks frequently adopt encoder-decoder frameworks trained with Dice or cross-entropy loss functions. These deep learning architectures and statistical metrics (Dice coefficient, intraclass correlation coefficients (ICCs), AUC) are standard, non-proprietary methods available for research use. Transformer-based and ensemble models demonstrated improved boundary accuracy and stability on heterogeneous MRI datasets. A minority of traditional methods (e.g., active contour and support vector machine-based classifiers) were retained as baselines but underperformed relative to modern architectures.

Thematic Analysis

Vertebral segmentation and labelling: Segmentation and labelling tasks represented the largest application group (12/20 studies; 60%). These models targeted vertebral body identification, spinal canal delineation, and disc segmentation. Reported Dice similarity coefficients ranged from 0.86 to 0.97, with mean intersection-over-union values exceeding 0.90 in several CT-based studies. Fully automated pipelines, particularly 3D CNNs and hybrid rule-based architectures, achieved reliable multi-level vertebral identification across cervical, thoracic, and lumbar regions. Transformer-enhanced segmentation models yielded the highest consistency on MRI-based datasets, improving delineation of soft-tissue boundaries.

Fracture detection and classification: Five studies (25%) focused on detecting vertebral compression fractures, typically using CNN-based detection frameworks such as YOLOv5 or EfficientNet. Reported diagnostic performance included AUCs ranging from 0.91 to 0.95, specificity above 90%, and sensitivity between 85% and 93%. Performance generally matched that of musculoskeletal radiologists in retrospective test sets. However, most models were limited to the lumbar spine and lacked external generalisation, with only one multicentre evaluation identified.

Automated morphometric and alignment measurement: Three studies (15%) developed algorithms for spinal alignment and morphometric measurement. These included CNN-based or regression models trained to automatically calculate parameters such as Cobb angle, pelvic tilt, sacral slope, and sagittal vertical axis. Agreement with expert measurement was excellent, with ICCs between 0.93 and 0.98 and mean absolute errors <2°. Such systems demonstrated potential for standardised, rapid assessment of scoliosis and sagittal balance. A subset of these studies further explored 3D reconstruction and postoperative assessment of implant alignment, enabling near real-time measurement from biplanar radiographs or CT series.

Soft tissue and pathology assessment: Three studies applied deep learning to paraspinal muscle segmentation, disc degeneration grading, and spinal canal stenosis detection using MRI. Reported Dice coefficients were 0.89-0.94, and classification accuracies for disc or stenosis grading reached 85-92%. While technical feasibility was high, dataset heterogeneity and limited sample sizes restricted generalisability.

Performance Metrics and Validation

Across all studies, technical performance metrics were strong but variably reported. Key metrics included Dice coefficients from 0.86 to 0.97 for segmentation, AUCs of 0.91 to 0.95 for fracture detection, and ICCs ranging from 0.93 to 0.98 for measurement tasks. However, only four studies (20%) performed external validation on independent datasets, and none included prospective evaluation or real-world clinical testing. Computational efficiency and inference times were variably reported, with most achieving sub-second inference per image on modern GPUs.

Methodological Quality and Risk of Bias

Across the 20 studies, most exhibited a moderate-to-high risk of bias owing to single-centre retrospective design, limited dataset diversity, and limited independent external validation. Only a small subset referenced multicentre cohorts or public datasets, and inter-rater reliability was infrequently reported. Preclinical/simulation settings were standard in workflow and registration research, further limiting clinical generalisability. Although reported accuracies and Dice coefficients were high, the methodological reporting seldom detailed safeguards against data leakage or overfitting, and reproducibility artefacts (e.g., open data/code) were rarely provided.

Discussion

This scoping review demonstrates the expanding role of computer vision in spinal orthopaedics, with consistently strong technical performance across segmentation, labelling, and fracture detection tasks. Segmentation remains the foundation of much of this work, reflecting the anatomical complexity of the spine and the centrality of vertebral and disc delineation to downstream applications. Dice scores exceeding 0.9 across multiple studies indicate excellent agreement with expert annotation, suggesting that vertebral segmentation on high-quality CT and MRI is approaching routine technical reliability. As a result, research focus is increasingly shifting from basic segmentation toward its applications, including 3D planning, patient-specific implant design, and biomechanical modelling.

Automated landmark detection for spinopelvic parameters also shows considerable promise, offering a pathway to more reproducible alignment assessment, a key determinant of postoperative outcomes. Fracture detection tools, particularly those applied to CT and MRI, demonstrate performance similar to that of musculoskeletal radiologists in retrospective testing. However, robust solutions for standard radiographs remain limited; the challenges of variable positioning, superimposed structures, and inconsistent image quality continue to restrict their reliability. MRI-based systems for grading disc degeneration and assessing stenosis are emerging but remain at an early stage of development.

The methodological appraisal highlights that technical excellence does not necessarily translate to a low risk of bias. The main limitations include reliance on single-centre datasets, inconsistent descriptions of ground-truth generation, and limited external validation, especially for registration and workflow-based studies. Most studies were retrospective, with minimal reporting of inter-rater agreement, calibration, or reproducibility measures. These limitations mirror challenges in other areas of musculoskeletal imaging and represent the major barrier to clinical adoption. Addressing them will require multicentre data curation, transparent reporting, independent external testing, and alignment with CLAIM recommendations for data documentation, annotation, and code availability.

A notable deficiency across the literature is the lack of prospective evaluation. None of the included studies assessed whether computer vision tools improve diagnostic accuracy, streamline surgical planning, or influence patient outcomes. Demonstrating accuracy is insufficient; evidence of clinical usefulness is required for regulatory approval and widespread integration. Prospective trials assessing outcomes such as operative efficiency, complication rates, and patient-reported outcome measures are essential for moving from proof-of-concept to clinically actionable systems.

There remains a significant opportunity for expanding beyond descriptive tools into prognostic modelling. Current systems predominantly identify or measure features within an image. A future generation of models could predict clinically relevant outcomes, such as the likelihood of vertebral collapse after a compression fracture or the risk of adjacent segment disease following fusion. Integrating imaging features with longitudinal clinical data offers an avenue for more personalised decision-making.

As computer vision workflows converge with navigation, robotics, and augmented reality, intra-operative decision support will likely become a major area of development [4]. Examples include real-time guidance for pedicle screw placement, automated warnings regarding neural proximity, and predictive modelling of postoperative alignment. For impactful implementation, these systems must integrate seamlessly into the operating theatre without increasing cognitive load or operative time. The human-computer interface, including how surgeons visualise and act on algorithmic outputs, remains an under-explored but essential area.

This review also identifies broader barriers to progress. High-quality labelled datasets remain the principal bottleneck. Establishing reliable ground truth is especially difficult for subjective tasks such as stenosis grading, where expert disagreement is common [40]. Linking imaging data to surgical findings and patient-reported outcomes will be essential for more clinically meaningful model training. Emerging approaches, such as self-supervised and federated learning, may help address data scarcity while preserving patient privacy and institutional data governance.

Practical, economic, and ethical considerations must also be addressed. Implementing these systems requires robust IT infrastructure, integration with radiological software and electronic patient records, and clear evidence of cost-effectiveness. Regulatory considerations, including accountability when algorithmic outputs influence clinical decisions, will shape how these tools are deployed in clinical environments. The field has made clear technical progress, but the work required for clinical translation, including validation, interpretability, and workflow integration, remains substantial [41].

## Conclusions

Computer vision has shown promising technical capability in spinal imaging, particularly in vertebral labelling and fracture detection. Deep learning models consistently achieve high accuracy and agreement with humans across CT and MRI modalities. However, clinical validation remains limited, and single-centre data and a lack of prospective studies limit implementation into practice. Future research should focus on generalisable, interpretable models that integrate seamlessly into orthopaedic and neurosurgical workflows.
